# Supporting Maternal Efforts to Provide Optimal Infant Nutrition in the Postpartum Setting^☆^

**DOI:** 10.1016/j.advnut.2024.100183

**Published:** 2024-02-02

**Authors:** Tina Gartley, Joel Bass, Ronald Kleinman

**Affiliations:** 1 Department of Pediatrics, Newton-Wellesley Hospital, Newton, MA, United States; 2 Department of Pediatrics, Massachusetts General Hospital, Boston, MA, United States

**Keywords:** breastfeeding, formula feeding, baby friendly, lactation support, safe sleep, birth hospitalization

## Abstract

Supporting optimal newborn nutrition and the positive maternal–infant relationship while encouraging safe sleep practices are essential components of maternal and newborn care in the hospital setting following birth. Breastfeeding is widely recognized as the best practice to support the nutritional needs and well-being of the infant, and recommendations have been developed by the WHO, the American Academy of Pediatrics (AAP), and the United States Centers for Disease Control to encourage and successfully support breastfeeding efforts before hospital discharge. The 10 Steps to Successful Breastfeeding, developed and promoted by the WHO, form the basis of the Baby-Friendly Hospital Initiative (BFHI) and have become the international framework for public health initiatives to promote breastfeeding. An evaluation of hospital performance implementing the 10 steps through the process of “Baby-Friendly Designation” (BFD) has been suggested by many breastfeeding advocates as the optimal pathway to attain the goals of the BFHI. However, the WHO has recognized that BFD may not be an appropriate goal in all settings, and indicated, as part of their updated 2018 guidance, that “facilities may make changes in their policies and procedures to obtain the designation, but these changes are not always sustainable, especially when there are no regular monitoring systems in place.” In addition, unintended associated issues regarding newborn safety and maternal dissatisfaction with some of the 10 steps have emerged. This perspective discusses the challenges faced by hospitals attempting to implement the BFHI 10 steps and suggests potential solutions to make progress in those efforts with or without BFD and also the efforts needed to support formula feedings when appropriate.

**Statement of Significance**

There is a critical need to understand and support hospital practices that will promote optimal newborn nutrition and safety following birth. Implementing the 10 steps of the BFHI is an important framework in this regard, to implement practices that lead to successful initiation of breastfeeding before hospital discharge, promote safe sleep practices related to breastfeeding and respecting maternal health and well-being. It is important to recognize that this can be accomplished without formal BFD. Although exclusive breastfeeding for approximately the first 6 mo of life is the standard for infant nutrition, some families may choose alternative methods of feeding. It is therefore incumbent on health care providers to provide education and support for all appropriate feeding choices.

## Introduction

**FIGURE 1:**
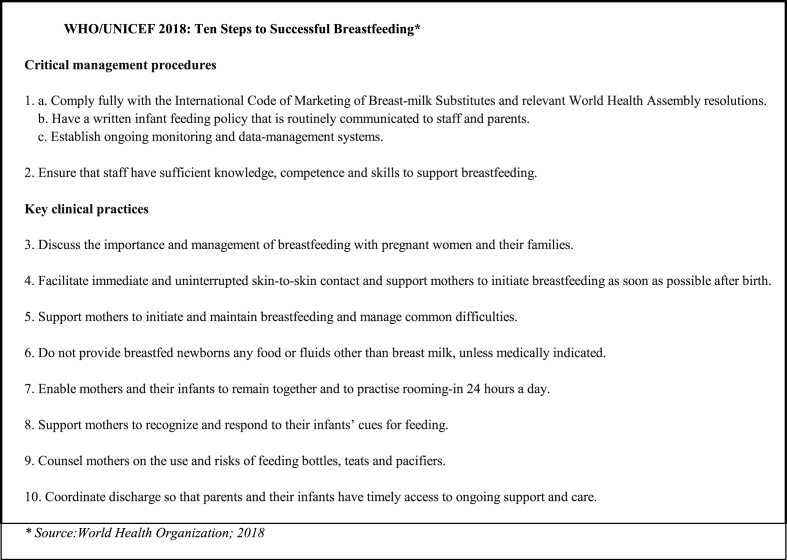
WHO and UNICEF 2018: Ten steps to successful breastfeeding. Adapted from reference [1] with permission. Protecting, promoting and supporting breastfeeding in facilities providing maternity and newborn services: implementing the revised Baby-friendly Hospital Initiative 2018. Geneva: World Health Organization and the United Nations Children’s Fund (UNICEF), 2018. Licence: CC BY-NC-SA 3.0 IGO.

In a WHO and UNICEF joint statement in 1989 the 10 Steps to Successful Breastfeeding (10 steps) were officially enumerated, as was the special role of maternity services to protect, promote, and support breastfeeding based on them [1]. The following year in Florence, Italy, the Innocenti Declaration on the Protection, Promotion, and Support of Breastfeeding set an international agenda with specific targets for action, which included universal adoption of the 10 steps [2]. The WHO and UNICEF developed the Baby-Friendly Hospital Initiative (BFHI) in 1991 [3], which has most recently been updated in 2018 and includes some revisions of the 10 steps (Figure 1) [4].

Multiple nations have developed processes to officially designate maternity services as “baby friendly.” In the United States, Baby-Friendly United States of America (BFUSA) is the accrediting body for hospitals to achieve designation as “baby friendly.” In 2011 the United States Surgeon General called for hospitals to accelerate the implementation of the BFHI [5]. The American Academy of Pediatrics (AAP) has consistently supported the implementation of the WHO BFHI 10 steps but has not endorsed universal Baby-Friendly Designation (BFD) [6]. This is an important distinction, as it allows for flexibility in the implementation of breastfeeding–supportive practices based on local needs and resources.

Over the years, concerns began to emerge concerning issues of maternal–infant well-being related to the rigid implementation of 10-step practices and BFD [7,8]. These include newborn falls associated with inadvertent co-bedding when rooming-in [9,10] and sudden unexpected postnatal collapse associated with unmonitored skin-to-skin care [11,12]. Newborn jaundice and dehydration have been associated with exclusive breastfeeding [[13], [14], [15]] and the WHO notes that late preterm infants are at greatest risk of these complications [1]. In addition, concerns in the medical literature have also been raised about the maternal experience in relation to their individual feeding and childcare decisions [16,17].

In response to these issues, the AAP issued guidance related to safe sleep and skin-to-skin care during birth hospitalization [11], the WHO significantly modified its BFHI guidelines [4,18] and BFUSA subsequently modified its designation criteria [19]. In addition, the United States Centers for Disease Control (CDC) modified its Maternity Practices and Infant Nutrition and Care survey [20], and the Joint Commission issued advisories on newborn falls [10] along with a new perinatal measure regarding unexpected death in term newborns [21]. The following discussion reviews the current challenges faced by hospitals attempting to support breastfeeding and suggest potential solutions to make progress in those efforts, as well as methods to support formula feeding when appropriate.

## AAP Policy Update

The AAP updated its guidance on breastfeeding in 2022 with the policy statement: Breastfeeding and the Use of Human Milk [22]. This updated statement continues to call on pediatricians to promote and support infant nutrition with breastfeeding and human milk as the normative standard. One of the most notable changes was the guidance to support continued breastfeeding for 2 y and beyond as mutually desired by mother and child. In conjunction with this, the AAP statement calls for the implementation of workplace and public policies that support families in sustaining breastfeeding and that national breastfeeding data should be collected through the first 2 y of life with attention to evaluating disparities in breastfeeding rates across racial, ethnic, and socioeconomic demographics.

The AAP revised policy statement on breastfeeding also includes an associated technical report [23] that updates the evidence for the statement and serves as a reference for other AAP policies that address breastfeeding. The AAP continues to recommend that hospitals implement breastfeeding–supportive practices as outlined in the WHO 10 steps (Figure 1) with some important caveats. The policy statement notes the importance of supporting breastfeeding with attention to unsafe practices, as outlined in the 2016 AAP policy on safe skin-to-skin care [11]. The technical report while recommending breastfeeding as one strategy to prevent sudden infant death syndrome also cites evidence that pacifier use, an evidence-based sudden infant death syndrome prevention strategy [24], before and after lactation is established, does not reduce the duration of breastfeeding for ≤4 mo following birth [23].

Another important update in the policy statement is information about the variation in the timing of the establishment of an adequate breastmilk volume to support the infant and an outline of maternal risk factors for delayed lactogenesis stage II, along with infant characteristics that are associated with inadequate milk or fluid intake. This allows health care providers to anticipate and recognize mother–infant dyads that need individualized support for lactation and breastfeeding. The policy also highlights the importance of supporting parents as they make informed decisions about feeding by stating that “the parental feeding decision should be fully supported, without pressure or guilt by any member of the health care team” and that “exclusive or any breastfeeding is not always possible, despite the best of intentions, and these mothers and families need special support to overcome the disappointment that may accompany breastfeeding difficulties” [22].

## Public Health Policy Update

Multiple organizations that provide guidance on improving the care of mothers and young infants have made recent revisions to their policies to firmly establish that breastfeeding practices must incorporate education concerning NIH safe sleep principles [24] and that providers need to be supportive of the infant feeding choices that mothers and families make. The CDC has also outlined strategies to implement breastfeeding support more safely [25]. These are reflected in the amended CDC Maternity Practices in Infant Nutrition and Care survey [20], which now includes questions regarding observation of the mother and infant by attendant staff during the first 2 h of life and maternal education to prevent newborn adverse events associated with maternal sleep and co-bedding while rooming-in.

The WHO’s revised implementation guidance [1] has also included specific wording to address the need for frequent staff assessments when implementing skin-to-skin care (step 4) and rooming-in (step 7). Following this, BFUSA adapted their implementation guidance to recommend the importance of staff vigilance regarding infant safety and distress during skin-to-skin practice in the immediate newborn period [19]. They currently advise the integration of safe sleep messaging with lactation education, with modeling of safe infant positioning and assessment of maternal risk factors for fatigue so that rooming-in can be implemented as safely as possible. BFUSA specifically cites AAP policy [11] as a source for facilities to consult for additional guidance in safely implementing breastfeeding–supportive hospital practices. BFUSA also includes extensive discussion of techniques for counseling and states that the “aim of breastfeeding counseling is to empower women to breastfeed, while respecting their personal situations and wishes” [19].

## Maternal Support for Newborn Nutrition During Birth Hospitalization

The changes in AAP and public health policies summarized above have provided a pathway to safely and effectively guide the efforts of postpartum units to promote and support breastfeeding. Exclusive breastfeeding for approximately the first 6 mo of life provides ideal nutrition for healthy full-term infants and is recommended by the WHO and the AAP [4,22]. In addition, the new 2030 Healthy People Goals now focus on long-term breastfeeding, exclusive breastfeeding at 6 mo, and any breastfeeding at 12 mo [26]. As the CDC breastfeeding report card data demonstrated, successful breastfeeding initiation during the hospital stay is critical to long-term breastfeeding success [27].

The updated WHO implementation guidance introduced many changes that have facilitated the efforts of hospital maternity services to help mothers safely initiate and sustain breastfeeding [1]. As designation is inconsistent and difficult to sustain, universal BFD is no longer recommended by the WHO [1]. Brodribb et al. [28] concluded that “when breastfeeding*-*initiation rates are high and evidence-based practices that support breastfeeding are common within the hospital environment, BFHI accreditation per se has little effect on both exclusive or any breastfeeding rates.” Although BFD is effective at attaining exclusive breastfeeding in hospital [5], an analysis of state-level data from all 50 states of the United States and territories demonstrated that the prevalence of breastfeeding initiation in a state rather than the percentage of BFD hospitals in that state is strongly associated with continued breastfeeding after discharge [27].

The details in the new WHO Evidence and Implementation documents [1,4] provide a framework that hospitals can follow as they develop their own institutional policies. As an example, although step 6 still recommends that breastfed newborns should not be provided any food or fluids other than human milk unless medically indicated, the new guidance does recognize that providing supplemental infant formula in the early days of life does not affect successful breastfeeding at discharge [4]. This new flexibility regarding supplemental feedings is an important advancement. When exclusive breastfeeding is enforced at night in the context of maternal exhaustion, resulting in unsafe sleep, including unintentional co-bedding, has been the source of related safety issues [7,11,29] and associated maternal dissatisfaction [4].

Medical indications for judicious use of supplementation include hypoglycemia, prematurity, and delayed lactogenesis associated with inadequate infant intake. The use of the Newborn Early Weight Loss Tool [30], which focuses on the percentile loss rather than the arbitrary 5% and 10% levels traditionally used, allows for a more nuanced determination of weight loss requiring supplementation.

AAP policy [22] and a recent review [31] have delineated the common maternal conditions that can delay the onset of full milk supply, including maternal obesity, maternal diabetes mellitus, hypertension in pregnancy, preterm labor, cesarean delivery, and delivery complications. Estimates in United States populations for delayed lactogenesis stage II are 35%–44% of primiparous and ∼19% of multiparous females [32,33]. Multiple studies have examined the effect of providing early limited formula from the perspective of infant readmissions, breastfeeding outcomes, and effect on the infant gut microbiome and have not demonstrated any consistent long-standing negative consequences [[34], [35], [36], [37]]. With the realization that many infants will need supplementation in the early days of life either due to medical necessity or maternal choice, an estimated 20% of hospitals are offering donor breast milk in their well-infant nurseries [[38], [39], [40]]. The benefit of donor breast milk in the preterm population is well established. In contrast, there is insufficient evidence to demonstrate the benefit of providing supplemental donor human milk as opposed to infant formula to the healthy term infant. This will be an important area of future research.

For formula-fed infants, clinicians need to provide information about safe preparation and recommended volume progression. Providing guidance not only ensures appropriate nutrition for infants for whom formula is a component of their diet but also allows families to feel supported by their health care provider. Formula preparation using powder requires clean technique with attention to boiling water for an appropriate length of time to minimize the chance of contamination [41,42]. Studies demonstrate that families do not consistently prepare infant formula correctly [43,44]. In the Infant Feeding Practices Survey II [32], only 20% of respondents reported boiling water when preparing formula. In conjunction with this, only 12%, reported being instructed by a health care provider on how to prepare formula. Families are also increasingly using non–FDA-approved formulas purchased from outside the United States that they perceive to be healthier for their infants and which may pose challenges with preparation directions, expiration information, and communication about potential recalls not being readily available in English [45]. In addition, many of these products are purchased from third-party vendors and safety of storage and handling cannot be ensured.

Maternity care centers need to be able to provide support based on a female’s individual circumstances that influence her infant feeding decisions. Females can choose from a variety of feeding plans based on their specific circumstances. This may include “pump and feed” and partial breastfeeding. Females with exclusive breastfeeding as their priority, should be provided the 10-step evidence-based support to promote breastfeeding initiation and exclusivity. For females who choose mixed feeding or exclusive pumping [46,47], support should be provided to ensure establishment of breastmilk supply. Those females who cannot or choose not to breastfeed should be provided specialized education on safe formula preparation. When all mother–infant dyads leave the hospital, they need individualized follow-up to support their feeding plans in the early weeks and months of life.

Implementing patient-centered care, focusing on sharing evidence-based information about infant feeding and allowing females to make individual decisions with nonjudgmental support from health care providers is critically important. The negative experiences that mothers cite with the BFHI in the United Kingdom were recently described in a systematic review by Fallon et al. [48]. In the United States, the commitment and difficulties associated with breastfeeding have sometimes been perceived as a threat to mother’s freedom and independence [5]. Providing breastfeeding guidance and support that promotes maternal self-efficacy and confidence is a key component of successful educational efforts [49].

## Support Beyond Birth Hospitalization

Since its inception, the WHO BFHI has recognized that optimal breastfeeding outcomes can be achieved when educational efforts begin prenatally, are implemented postpartum and continued after hospital discharge [2]. This can be accomplished by incorporating breastfeeding education in prenatal classes and fostering the establishment of breastfeeding support groups that mothers can be referred to on discharge. Maternity services can leverage their impact by incorporating lactation education and support throughout the continuum of newborn care.

It is also well recognized that parents use the Internet for health information and this information is not consistently reviewed with their pediatrician [50,51]. Females are increasingly using social media for information about breastfeeding [52,53]. Innovative ways of delivering support include telelactation services [54,55] and other online support groups. This will be an important area of research for clinicians to be engaged in to help patients meet their individual feeding goals. The new AAP policy [22] also calls for pediatricians to become involved in advocating for local and federal policies that protect breastfeeding and support the efforts of families in feeding their infants. Their participation could have a positive influence on the future direction of health policy efforts to strengthen system-level support for breastfeeding initiatives [56].

In conclusion, the current state of AAP and public policy positions supports the implementation of the 10 steps of the BFHI during the postpartum hospital stay. Although some institutions may decide to pursue BFD, BFHI principles can be successfully accomplished independent of designation. The overarching goal in all cases should be to support and educate all families in a manner that ensures optimal nutrition to support growth, development, and health in a safe and effective way.

### Author contributions

The authors’ responsibilities were as follows – TG: conceptualization, writing original draft, review, and editing; JB: conceptualization, writing original draft, review, and editing; RK: conceptualization, review and editing; and all authors have read and approved the final manuscript.

### Conflict of interest

RK reports a relationship with Arla Foods that includes: consulting or advisory; a relationship with General Mills Inc that includes: consulting or advisory; and a relationship with Ocean Spray Cranberries Inc that includes: consulting or advisory. The other authors report no conflicts of interest.

### Funding

The authors reported no funding received for this perspective.
